# High-resolution time series of *Pseudomonas aeruginosa *gene expression and rhamnolipid secretion through growth curve synchronization

**DOI:** 10.1186/1471-2180-11-140

**Published:** 2011-06-17

**Authors:** Dave van Ditmarsch, João B Xavier

**Affiliations:** 1 Computational Biology Program, Memorial Sloan-Kettering Cancer Center, 408 East 69th Street, New York NY, 10021-5604, USA

## Abstract

**Background:**

Online spectrophotometric measurements allow monitoring dynamic biological processes with high-time resolution. Contrastingly, numerous other methods require laborious treatment of samples and can only be carried out offline. Integrating both types of measurement would allow analyzing biological processes more comprehensively. A typical example of this problem is acquiring quantitative data on rhamnolipid secretion by the opportunistic pathogen *Pseudomonas aeruginosa*. *P. aeruginosa *cell growth can be measured by optical density (OD_600_) and gene expression can be measured using reporter fusions with a fluorescent protein, allowing high time resolution monitoring. However, measuring the secreted rhamnolipid biosurfactants requires laborious sample processing, which makes this an offline measurement.

**Results:**

Here, we propose a method to integrate growth curve data with endpoint measurements of secreted metabolites that is inspired by a model of exponential cell growth. If serial diluting an inoculum gives reproducible time series shifted in time, then time series of endpoint measurements can be reconstructed using calculated time shifts between dilutions. We illustrate the method using measured rhamnolipid secretion by *P. aeruginosa *as endpoint measurements and we integrate these measurements with high-resolution growth curves measured by OD_600 _and expression of rhamnolipid synthesis genes monitored using a reporter fusion. Two-fold serial dilution allowed integrating rhamnolipid measurements at a ~0.4 h^-1 ^frequency with high-time resolved data measured at a 6 h^-1 ^frequency. We show how this simple method can be used in combination with mutants lacking specific genes in the rhamnolipid synthesis or quorum sensing regulation to acquire rich dynamic data on *P. aeruginosa *virulence regulation. Additionally, the linear relation between the ratio of inocula and the time-shift between curves produces high-precision measurements of maximum specific growth rates, which were determined with a precision of ~5.4%.

**Conclusions:**

Growth curve synchronization allows integration of rich time-resolved data with endpoint measurements to produce time-resolved quantitative measurements. Such data can be valuable to unveil the dynamic regulation of virulence in *P. aeruginosa*. More generally, growth curve synchronization can be applied to many biological systems thus helping to overcome a key obstacle in dynamic regulation: the scarceness of quantitative time-resolved data.

## Background

Spectrophotometric measurements are ubiquitous for quantitative analyses of dynamic biological processes. In contrast, many other useful measurements require laborious sample treatment that may include separation or extractions, colorimetric reactions, electrophoresis as well as many other biochemical analyses. These latter measurements are generally done as endpoint or offline measurements. As opposed to the high temporal resolution of online measurements, offline measurements cannot generally be used to monitor a dynamic process with the same frequency. Furthermore, when the analyses require sample destruction then the offline method can only be used for endpoint measurements. This raises the question whether offline measurements can be integrated with high-resolution online measurements for a more comprehensive examination of biological processes.

Here, we propose a simple method to integrate cell growth data monitored at high temporal resolution with endpoint measurements of secreted metabolites that require offline sample treatment. The method takes advantage of the exponential growth of bacterial cultures [1]. For typical cell cultures, where growth curves are highly reproducible, the serial dilution of an inoculum will lead to growth curves that are shifted in time. The time-shift is the combination of a period of cell adaptation (the "lag" phase [1]) and the time it takes for the culture to grow to detectable values of cell density. The total shift is longer in cultures started from lower concentrations because it takes more cell divisions to reach the detectable cell density. If the lag period is independent of cell density, then the growth curves are only shifted in time due to the differences in initial density and growth curves can be synchronized *a posteriori *by calculating the time-shift that maximizes the overlap between them. Performing endpoint assays on cultures started at the same time but with different dilutions will then allow a time series of measurements to be reconstructed from the calculated time-shifts.

This approach of growth curve synchronization has several advantages over sampling a system at different times. Firstly, the endpoint measurements can all be performed at the same time, thereby decreasing experimental variability. Secondly, efficiency will be improved compared to processing multiple samples at different times. Thirdly, no invasive sampling is necessary and the method requires no constant vigilance or presence. Finally, as we discuss throughout the paper, it allows measuring the division rate of cells directly from optical density with very high precision.

We exemplify the growth curve synchronization method by analyzing rhamnolipid secretion by the bacterium *Pseudomonas aeruginosa*. *P. aeruginosa *is an opportunistic human pathogen found in long-term, often terminal, infections in cystic fibrosis patients and various nosocomial infections occurring in immunocompromized patients [2-9]. Rhamnolipids are among the predominant virulence factors of *P. aeruginosa *[9,10]. These glycolipid surfactants are involved in the formation and maintenance of biofilms, cytolysis of polymorphonuclear leukocytes (PMNs) and swarming motility ([8,11]; reviewed in [12]). Their synthesis is regulated by quorum sensing, a mechanism for cell density-dependent gene regulation. As such, rhamnolipid secretion in *P. aeruginosa *is a valuable model system to investigate how pathogenic bacteria coordinate population-wide traits at the molecular level [13].

The rhamnolipid quorum-sensing regulation consists of at least two hierarchical systems governed by two different autoinducers [14-23]. These two systems, called *rhl *and *las*, share a common motif. An autoinducer synthase (RhlI and LasI) synthesizes the autoinducer (*N*-butyryl-L-homoserine lactone or C_4_-HSL and *N*-(3-oxododecanoyl)-L-homoserine lactone or 3O-C_12_-HSL), which binds to its cognate transcription factor (RhlR and LasR) that, in turn, up-regulates the autoinducer synthase in a positive feedback. LasR controls expression of RhlR, and thereby the *las *system is hierarchically above *rhl*. The *rhl *system induces expression of *rhlAB*, resulting in rhamnolipid production [24]. In spite of this knowledge, the rhamnolipid system has puzzled microbiologists because it does not behave like the paradigm of quorum sensing [13,25,26]. In either *rhlI*^- ^or *lasI*^- ^bacteria, adding autoinducers to the growth media does not induce rhamnolipid secretion from the outset of the culture, indicating there is at least one other factor regulating *rhlAB *expression [13].

Here we illustrate our growth curve synchronization method by integrating high-resolution spectrophotometric measurements of cell density and gene expression with endpoint rhamnolipid quantification to produce multi-measurement time series of the latter. We monitor cell density by optical density at 600 nm, *rhlAB *expression using a GFP reporter fusion under the control of the *rhlAB *promoter (P*_rhlAB_*::*gfp*) and secreted rhamnose using the sulfuric acid anthrone assay [27]. We also illustrate how this simple method can be used in combination with isogenic mutants lacking specific genes in the rhamnolipid synthesis or quorum sensing regulation to shed new light on the regulation of *P. aeruginosa *virulence.

## Methods

All chemicals were acquired from Fisher Scientific (Waltham, MA) unless specified.

### Bacterial strains

The strains used in this study are listed in Table 1. We used *Pseudomonas aeruginosa *PA14 as the parental strain for all further constructions. A published GFP reporter fusion [25] was cloned into wild-type PA14 cells (*P. aeruginosa *PA14 P*_rhlAB_*::*gfp*; strain denoted as WT). A clean rhamnolipid-deficient deletion mutant (*ΔrhlA *[13]) was used to construct a strain with *rhlAB *under the control of the arabinose-inducible P_BAD _promoter (*P. aeruginosa *PA14 *ΔrhlA*/P_BAD_::*rhlAB*; strain denoted as IND, the inducible construct was described in [28]) as well as a GFP reporter fusion strain (*P. aeruginosa *PA14 *ΔrhlA*/P*_rhlAB_*::*gfp*; strain denoted as NEG). The quorum sensing signal negative strain (*rhlI*^-^) is a transposon insertion obtained from the PA14 non-redundant mutant library [29]. The GFP reporter fusion was also cloned into this strain, yielding *P. aeruginosa *PA14 *rhlI*^-^/P*_rhlAB_*::*gfp *(strain denoted as QSN).

**Table 1:** *Pseudomonas aeruginosa *strains used in this study

Strain	Genotype	Description	Reference or origin
WT	PA14 P*_rhlAB_*::*gfp*	The wild-type background with a P*_rhlAB_*::*gfp *reporter fusion	[13,25]
NEG	PA14 *ΔrhlA*/P*_rhlAB_*::*gfp*	Same as WT but with rhamnolipid synthesis gene *rhlA *deleted.	This study
QSN	PA14 *rhlI*^-^/P*_rhlAB_*::*gfp*	Same as WT but with a transposon knockout of *rhlI *gene for autoinducer synthase.	This study
IND	PA14 *ΔrhlA*/P_BAD_::*rhlAB*	Strain with rhamnolipid synthesis genes *rhlAB *regulated by an L-arabinose inducible promoter.	[13]

### Media and growth conditions

Overnight starter cultures were inoculated directly from glycerol stocks into 3 ml of LB Broth, Miller (EMD chemicals, Gibbstown, NJ) and incubated for 16-18 h at 37°C in a rotator shaker. Growth curve assays in microtiter plates were carried out in minimal synthetic media with the following composition: 64 g/L of Na_2_HPO_4_.7H_2_O, 15 g/L of KH_2_PO_4_, 2.5 g/L of NaCl, 1 mM of MgCl_2_, 0.1 mM of CaCl_2_, 3 grams of carbon per liter in glycerol and 0.5 grams of nitrogen per liter in ammonium sulfate. When necessary, media were supplemented with either 0.5% (w/v) L-arabinose (MPBio, Solon, OH) or 5 μM *N*-butyryl-L-homoserine lactone (C_4_-HSL; Sigma-Aldrich, St. Louis, MO) to induce *rhlAB *expression in IND or to activate the quorum sensing conditions for QSN, respectively.

### Microtiter plate assays

Cells from overnight cultures were washed twice in 1 × phosphate-buffered saline (PBS). Each of the serial dilutions was then diluted into minimal synthetic media at the appropriate dilution ratio in 1.5 mL microcentrifuge tubes (OD_600 _of 0.0025 for the undiluted sample and twofold dilutions for each following sample). At the lowest densities even small numbers of bacterial cells sticking to the walls of the tubes will introduce high variability. This problem can be avoided by systematically vortexing the bacteria immediately before transferring to new tubes or to the microtiter plate where the growth will be measured. Growth assays were conducted in clear flat-bottom BD Falcon 96-well plates (BD Biosciences, San Jose, CA), containing 8 replicates of 150 μL per sample (or 4 replicates in the case of IND with and without C_4_-HSL). The plates were incubated at 37°C in a Tecan Infinite M1000 plate reader (Tecan US Inc., Durham, NC) set to "incubation mode" with orbital shaking of 4 mm amplitude. Optical density at 600 nm (OD_600_) and GFP fluorescence (λ_excitation _= 488 nm, λ_emission _= 525/40 nm) were measured every 10 minutes for the duration of the assay (32 h).

### Anthrone assay to quantify rhamnolipids

After each assay, the eight replicates of each sample were pooled together in a microcentrifuge tube. The cells were spun down at 7,000 rcf for 2 minutes. Pooling the replicates will lead to considerable foaming because of rhamnolipids in the supernatant. This foam contains a significant amount of rhamnolipids and must therefore be collected. 750 μL of the supernatant were transferred to a new microcentrifuge tube. Rhamnolipid extraction was then carried out twice via liquid-liquid extraction using 750 μL of chloroform:methanol at 2:1 (v:v) each time. When experiments had only four replicates we used a variation of this extraction protocol, transferring 500 μL of the supernatants and extracting with 500 μL of chloroform:methanol each time. The organic phases of both extractions were pooled and then evaporated to dryness in a Vacufuge Concentrator (Eppendorf, Hauppauge, NY) at 60°C. Each sample was subsequently re-suspended in 100 μL of pure methanol, so that the final rhamnolipid concentration is 7.5 × higher than in the initial culture (or 5 × for experiments with 4 replicates). Quadruplicate samples of 20 μL each were then prepared together with quadruplicate samples of an L-rhamnose (Indofine Chemical Company, Hillsborough, NJ) ladder in a Thermogrid 96-well PCR plate (Denville Scientific, Inc., Metuchen, NJ). The plate was put in iced water and 200 μL of anthrone (Alfa Aesar, Ward Hill, MA) solution (0.1% (w/v) in 70% (v/v) H_2_SO_4_) were added to each sample before heating the entire plate to 80°C for 30 minutes. At this point the degree of blueness indicates the amount of rhamnose in a sample. 200 μL of each sample were then transferred to a clear flat-bottom 96-well plate and the absorbance was measured at 630 nm. The absorbance levels were converted to rhamnose concentration using the rhamnose calibration values.

### Computational alignment of growth curves

All growth curve analysis and plotting was carried out in Matlab (the Mathworks, Inc., Natick, MA). An in-house algorithm (Additional files 1 and 2, with example data in Additional Files 3 and 4) was written for time shift calculations, which maximizes time-series overlap between all possible permutations of different growth curves by minimizing the error of the median values. The minimization routine uses the function *fminsearch *from the Matlab Optimization toolbox, which is a derivative-free method to search for minima of unconstrained multivariable functions. The time-shifts (τ) of the different curves were then used to recreate a time series of L-rhamnose quantifications.

## Results

### Mathematical model supporting the growth curve synchronization method

The range of inoculum densities that may be used for growth curve synchronization has both an upper and a lower limit. While one can determine these limits experimentally by testing whether the experiment works over a large range of values, the factors behind these constraints have the following straightforward theoretical explanation. The lower limit for initial cell density is set by small number statistics. If the inoculum is too dilute then there is a significant probability that some wells will not receive any cells. The probability of having empty wells can be calculated since the number of cells in the inoculum follows a Poisson distribution. For example, in the extreme case where an inoculum has an average of 1 cell per replicate, the probability of having at least one replicate among eight with zero cells is 97%. The upper limit for inoculum density, on the other hand, is determined by the carrying capacity of the growth media. In order to guarantee reproducibility between growth curves started from inocula at different densities, the differences between the initial cell densities must be negligible compared to the carrying capacity yet they must not suffer from the small number statistics.

Typical growth curves are subdivided into three phases [1]: a lag phase, an exponential phase and a stationary phase. The exponential phase starts when cells begin dividing at a constant rate, such that density increase follows  (*μ*_max_ is called the maximum specific growth rate.) The stationary phase starts when growth slows down as the system approaches carrying capacity. Decreasing growth rate can attributed to nutrient depletion, accumulation of metabolic waste or density-dependent growth regulation, among other things [1,30-35]. Here, we formulate a mathematical model assuming that growth limitation is due to nutrient depletion, but the same analysis can be applied to any other limiting factor. Without loss of generality we use Monod's equation [1] to model bacterial growth based on nutrient concentration (*N*)

where *K_N _*is the half-saturation constant. The nutrient concentration, initially *N*_0_, decreases as a function of cell growth and the yield (*Y*) such that at a time *t *it has the value

The maximum cell density reached (i.e. the carrying capacity) is 

Growth starts to slow down as nutrient levels decrease to levels close to the half-saturation constant, *K_N_*. For example, when *N*(*t*) is equal to 20 × *K_N_*, the growth rate is theoretically ~95% of *μ*_max_. Such a 5% decrease is typically undetectable by optical density measurements [36]. Therefore, in theory, as long as the initial cell density is *X*_0 _<<*Y *× 20 × *K_N_*, variations in the inoculum density have negligible impact on growth curve reproducibility. This therefore sets an upper limit to the inoculum density.

Besides the lower and upper limits of inoculum density, another important condition for the growth curve synchronization is that the lag phase must be independent of inoculum concentration. We can confirm if this is true by testing whether the time shift (τ) between growth curves starting from cell densities *X*_1 _and X_2 _(where *X*_2 _>*X*_1_) obeys the following relationship

Below, we show how we tested this condition empirically for all growth curves aligned by calculating the linear regression between τ and ln (*X*_2_/*X*_1_).

### Application to virulence factor secretion by *Pseudomonas aeruginosa*

**Figure 1:**
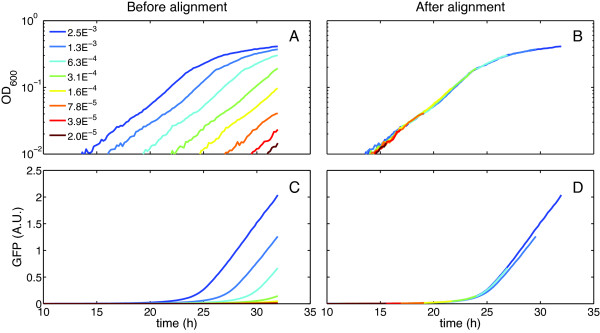
***Alignment of growth curves and GFP expression in WT strain***. A) Median growth curves constructed from 8 replicates of cultures inoculated between 0.0025 OD_600 _(dark blue) and 2 × 10^-5 ^OD_600 _(dark red). B) Growth curve alignment for the median growth curves. C) Median GFP expression curves, constructed from the same samples as the growth curves. D) GFP curves aligned using the time-shift calculated from the OD_600 _alignment.

**Figure 2:**
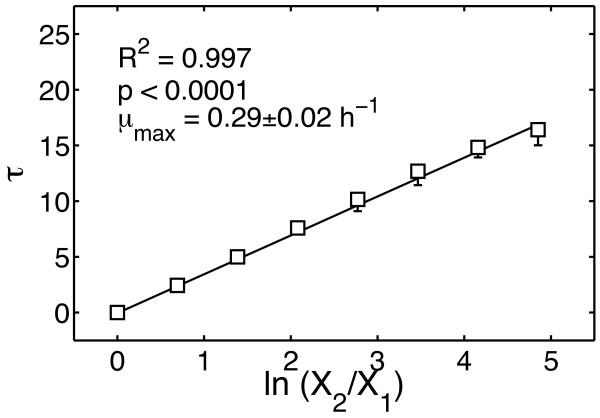
***Determining the reproducibility of the lag phase in WT cells***. If the mathematical assumption τ = (1/*μ*_max_) ln (X_2_/X_1_) is correct, then τ as a function of ln (X_2_/X_1_) should yield a straight line with a slope of 1/*μ*_max_. τ shows a correlation to ln (X_2_/X_1_) with an R^2 ^of 0.997 (p < 0.0001) and a *μ*_max _of 0.29 ± 0.02 h^-1 ^for WT. The median and range over three independent experiments are plotted as black squares and error bars.

We used high-resolution OD_600 _curves of wild-type *P. aeruginosa *PA14 to demonstrate the growth curve synchronization method. The wild-type strain will be referred to as WT (see Table 1 for list of strains used). Figure 1 shows 8 growth curves obtained by serial dilution before (Figure 1A) and after alignment (Figure 1B). Although visual inspection shows the alignment was successful, we evaluated the quality of the alignment by plotting the time delays (τ) as a function of the log of the dilutions (Figure 2). For this case, we obtained R^2 ^= 0.996, which confirmed the alignment is appropriate and confirms that the lag phase is independent of inoculum density, which is a central requirement of our method. Figure 1C shows GFP expression measured for the same samples. GFP expression is under the control of the *rhlAB*-promoter, making GFP an indication of the expression of rhamnolipid synthesis genes. Figure 1D shows the alignment of GFP expression obtained using the time delays calculated from the original synchronization based on OD_600_. This alignment shows that gene expression monitored by a reporter protein can be synchronized using the same time-shift, without the need for a separate calculation, again supporting our theoretical model.

**Figure 3:**
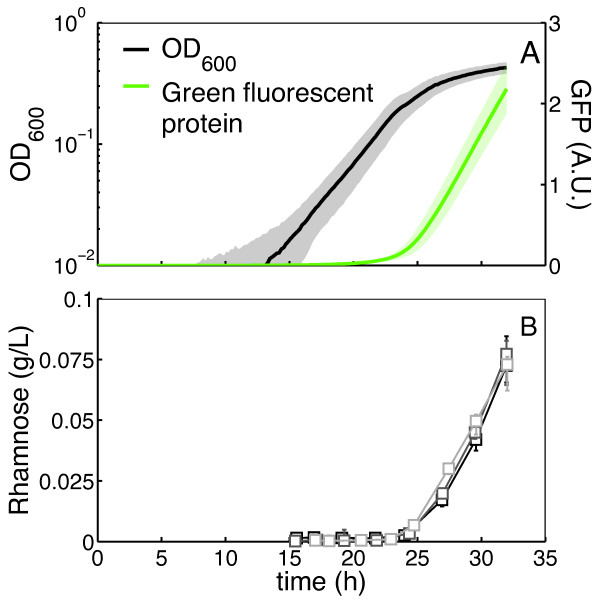
***Average growth, GFP expression and rhamnose secretion in WT cells***. A) Average growth of WT cells (black) with standard deviation (gray), inoculated at 0.0025 OD_600 _over three independent experiments. Average GFP expression (in arbitrary units), under the control of the PA01 rhlAB-promoter (green) with the standard deviation (light green) constructed from the same experiments. B) Time series of rhamnose secretion in WT from three independent experiments (grayscale squares). The time series were constructed using the calculated time-shifts from the respective experiments. For each rhamnose measurement, the median is plotted with the entire range of the measurements represented as error bars.

Figure 3A shows the average growth curve (OD_600_) and the average *rhlAB*-expression curve (by way of a GFP reporter) of WT, with their respective standard deviations, reconstructed with data from three independent experiments. These reconstructions show that expression of rhamnolipid synthesis genes started only when the culture entered stationary phase, as observed previously in experiments with richer media [13,25]. We then used the calculated time shifts from the growth curve synchronizations to reconstruct time series of rhamnolipid secretion. The two-fold serial dilution used for preparation of the inocula produced a reconstructed time series with one rhamnolipid measurement approximately every ~2.5 h, which corresponds to a ~0.4 h^-1 ^frequency (Figure 3B). The reconstructed series also revealed that secreted rhamnose levels quickly follow the onset of GFP expression.

**Figure 4:**
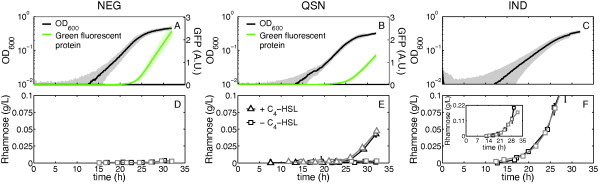
***Average growth curves, GFP expression and rhamnose secretion in strains NEG, QSN and IND***. A) Average growth of NEG cells (black) with standard deviation (gray), inoculated at 0.0025 OD_600 _over two independent experiments. Average GFP expression, under the control of the PA01 *rhlAB*-promoter (green) with the standard deviation (light green) constructed from the same experiments. B) Average growth of QSN cells in the presence of 5 μM C_4_-HSL (black) with standard deviation (gray), inoculated at 0.0025 OD_600 _over two independent experiments. Average GFP expression, under the control of the PA01 *rhlAB*-promoter (green) with the standard deviation (light green) constructed from the same experiments. C) Average growth curve of IND cells (black) with standard deviation (gray), inoculated at 0.0025 OD_600 _over two independent experiments. NEG is not a reporter fusion strain, so there is no GFP expression. D) No rhamnose is detectable in NEG in two independent experiments (black and gray). E) Rhamnose is undetectable in QSN in the absence of C_4_-HSL in two independent experiments (black and gray squares), but is reconstituted in the presence of C_4_-HSL (black and gray triangles). F) Rhamnose secretion in IND in two independent experiments (black and gray squares). The inset shows the complete range of rhamnose secretion in IND cells under our experimental settings.

**Figure 5:**
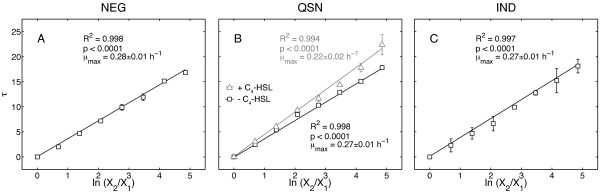
***Determination of the reproducibility of the lag phase in NEG, QSN and IND***. For NEG, τ shows a correlation to ln (X_2_/X_1_) with an R^2 ^of 0.998 (p < 0.0001) and a *μ*_max _of 0.28 ± 0.01 h^-1^. The median and range over two independent experiments are plotted as squares and error bars. For QSN in the absence of autoinducer, τ shows a correlation to ln (X_2_/X_1_) with an R^2 ^of 0.998 (p < 0.0001) and a *μ*_max _of 0.27 ± 0.01 h^-1^. In the presence of C4-HSL τ shows a correlation to ln (X_2_/X_1_) with an R^2 ^of 0.994 (p < 0.0001) and a *μ*_max _of 0.22 ± 0.02 h^-1^. The median and range over two independent experiments are plotted as black squares (without autoinducer) or gray triangles (with autoinducer) with their respective error bars. For IND, τ shows a correlation to ln (X_2_/X_1_) with an R^2 ^of 0.997 (p < 0.0001) and a *μ*_max _of 0.27 ± 0.01 h^-1^. The median and range over two independent experiments are plotted as squares and error bars.

Next, we performed the same experiment for an isogenic mutant lacking the gene *rhlA *(strain NEG) as a negative control (Figure 4A). As for WT, the growth curves aligned well (R^2 ^= 0.998, Figure 5A). An average growth curve and an average GFP expression curve were constructed, showing that NEG cells would still express the *rhlA *synthesis genes when entering the stationary phase if the gene was present (green curve in Figure 4A). As expected, rhamnolipid secretion was undetectable (Figure 4D).

We then used the same method for a signal-negative mutant, QSN, both in the absence and in the presence of autoinducer (C_4_-HSL) supplied in the media. Again, the growth curves aligned well for both conditions (Figure 5B; R^2 ^= 0.998 and R^2 ^= 0.994, respectively). As expected, the cells did not secrete rhamnolipids in the absence of C_4_-HSL (Figure 4E, gray and black squares), but the addition of 5 μM C_4_-HSL to the media restituted rhamnolipid production (Figure 4E, gray and black triangles). Importantly, although the amount of gene expression and rhamnolipid secretion in the presence of C_4_-HSL was lower than for WT both at the population- (Figure 2) and individual cell-level (as assessed by GFP divided by OD, data not shown), the timing remained the same (Figure 4B). This is consistent with previous observations that the time delay of the quorum sensing-controlled *rhlAB *operon in signal-positive *P. aeruginosa *is maintained even when the medium is complemented with high concentrations of autoinducers [13,25].

We then carried out experiments with an inducible strain (IND), which expresses *rhlAB *constitutively upon induction with L-arabinose. The purpose of this experiment was to provide a positive control showing that the only requirement for rhamnolipid secretion is the expression of *rhlAB *[24]. The growth curves for this strain also aligned well (R^2 ^= 0.997, Figure 5C). When IND was grown with 0.5% (w/v) L-arabinose, rhamnolipid production was detected much earlier, starting already in the exponential phase, instead of at the beginning of the stationary phase (Figure 4C and 4F).

### Time-shifts provide high precision measurement of growth rate

Since the relation τ = (1/*μ*_max_) ln (X_2_/X_1_) governs the time shift (τ) between different growth curves, τ can be plotted as a function of ln (X_2_/X_1_) yielding a straight line with a slope of 1/*μ*_max_. This allows calculating the maximum specific growth rate (*μ*_max_) from the growth curve synchronization. When performing this quantification, we observed that WT and NEG have comparable growth rates (Figures 3 and 5A; *μ*_max _= 0.29 ± 0.02 h^-1 ^versus μ_max _= 0.28 ± 0.01 h^-1^, respectively), which was already shown qualitatively in previous experiments with rich media based on casamino acids and in direct competition experiments [13]. QSN also showed growth rates comparable to WT in the absence of C_4_-HSL (Figure 5B, squares; *μ*_max _= 0.27 ± 0.01 h^-1^). However, when C_4_-HSL was added to the media, QSN grew markedly slower (Figure 5B, triangles; *μ*_max _= 0.22 ± 0.02 h^-1^). C_4_-HSL was solubilized in acetonitrile, but the addition of acetonitrile without autoinducer did not affect growth (data not shown). To the best of our knowledge, this effect has not been observed before. The addition of 0.5% L-arabinose to the growth media of IND did not affect their growth, as the growth rate was similar to WT cells (Figure 5C; *μ*_max _= 0.27 ± 0.01 h^-1^).

## Discussion

We introduced the method of growth curve synchronization for the *a posteriori *synchronization of high-resolution time series and integration of online spectrophotometric data with endpoint measurements. We demonstrated the method with growth curve data from the opportunistic human pathogen *Pseudomonas aeruginosa *PA14 and isogenic mutants. The quality of the growth-curve alignments was assessed by measuring the R^2^-values for the linear regression of the calculated time-shift (τ) versus the logarithm the inoculum (R^2 ^> 0.99 in all cases, Figures 3 and 5), a relationship that we formulated based on a simple mathematical model of exponentially growing cell cultures.

In addition to carrying out data integration, our method provides a high-precision measurement of maximum specific growth rate. Figures 3 and 5 show the maximum specific growth rates (μ_max_) measured from the slope of the τ vs. ln(X_2_/X_1_). The average error of these measurements evaluated from the regression was 5.4%. In the worst case, being QSN in the presence of C4-HSL (Figure 5B, triangles), the error was 9.1%. This precision is quite good for growth rates measured from optical density, approaching the 5% error reported for a high-precision bioluminescence-based method [36]. However, in contrast to a bioluminescence assay, our OD-based method does not require introduction of a constitutively expressed luciferase reporter or the use of an expensive bioluminescence-capable reader.

Besides serving as examples of the application of the growth curve synchronization method, the experiments reported here help to further clarify the regulation of rhamnolipid secretion in *P. aeruginosa*. The WT time series (Figure 2A) show, as before [13,25], that *rhlAB *promoter-controlled GFP was expressed at the onset of the stationary phase. Here we complement this observation by showing for the first time that the onset of rhamnolipid production follows the same timing as the gene expression using the reconstructed time series of rhamnolipid secretion (Figure 2B). This supports biochemical studies suggesting that expression of *rhlAB *is the main step controlling the start of rhamnolipid synthesis [24]. The strain with the reporter fusion in the *ΔrhlA *background (NEG) showed that up-regulation of the gene is still active and that cells would still produce rhamnolipids if *rhlA *was not deleted (Figure 4A and 4D). The fact that the timing and quantity of GFP expression for this strain (Figure 4A) resembles that of WT expression (Figure 2A) suggests that there is no feedback of biosurfactant synthesis on the expression of *rhlAB*. Our experiments also confirmed that cells lacking autoinducer synthesis (QSN) do not express *rhlAB *nor produce rhamnolipids in the absence of autoinducer (Figure 4E, black and gray squares). As expected, both *rhlAB *expression and rhamnolipid secretion were recovered when the autoinducer was supplied in the medium (Figure 4B and 4E, black and gray triangles). Interestingly, however, even in the presence of autoinducer in the medium *rhlAB *expression and rhamnolipid secretion were not constitutive but rather the delay until entry into the stationary phase (Figure 4B and 4E, triangles and [13,26,37]) that is characteristic of the wild-type was maintained. We then confirmed that it is, in fact, possible for *P. aeruginosa *to start rhamnolipid secretion earlier in growth by using an *rhlAB*-inducible strain (IND). With the level of inducer used (0.5% (w/v) L-arabinose) IND started rhamnolipid secretion already in the exponential phase of growth (Figure 4C and 4F). Taken together our observations further support that rhamnolipid secretion has additional regulation besides quorum sensing. Such regulation was recently proposed to be a molecular mechanism of metabolic prudence that stabilizes swarming motility against evolutionary 'cheaters' [13].

Our measurements are population averages even though systems biology is increasingly focusing on single-cell measurements. However, there is presently no method to measure rhamnose secretions in single cells. Nonetheless, reconstruction of distributions of single-cell gene expression is possible using reporter fusions either by fluorescence microscopy [38] or flow-cytometry [39]. Such single-cell measurements can be carried out offline and reconstructed into time series using our method of growth curve synchronization. The colorimetric anthrone assay used here measures the amount of rhamnose, which is an indication of the amount of rhamnolipids. This does not necessarily correspond linearly to the mass of rhamnolipids secreted. The rhamnolipids secreted by *P. aeruginosa *can have variable composition (reviewed in [12]) and rhamnolipids exist both in mono- and di-L-rhamnose forms. Methods such as thin layer chromatography, to distinguish the mono-L-rhamnose from di-L-rhamnose rhamnolipids, or mass spectrometry [40] allow more precise measurements. These analyses could be used to complement reconstructed time series and help further characterize the regulation of rhamnolipids, which are important virulence factors for *P. aeruginosa *[9,10]. In the long term, unveiling the molecular mechanisms regulating the timing and quantity of rhamnolipid secretion can lead to the rational development of new therapies that specifically target virulent secretions to fight *P. aeruginosa *infection.

Cell density in bacterial and other cell populations is often monitored by optical density at 600 nm (OD_600_), in spite of its inherent noisiness and limited dynamic range. For this reason, we chose to apply our method to time series of OD_600_. We envision that any other high-resolution time series data should be useable for aligning curves, including fluorescence or bioluminescence. The only requirement is that the calculated time delays and inoculum dilution must have a linear relationship for the range of inoculum concentrations used (Figures 2 and 5). The alignment method we used was an algorithm developed specifically for our purpose (code supplied as supporting material). Nevertheless, any other algorithm that aligns sets of growth curves and that determines concomitant time delays can in principle be used. We also tested our analysis by aligning the growth curves visually. Although the visual alignment gave acceptable results (not shown), an automated method using an unsupervised yet robust algorithm such as the one provided here is preferable for speed and consistency (manual alignment is possible through Additional File 5).

The method introduced here can potentially be applied to many other experimental problems that have exponentially growing cultures and where the integration of online and offline measurements is desired. Besides the growth of *P. aeruginosa *and its rhamnolipid secretion, another example is indole production by altruistic bacteria [41]. Indole was found to be important for antibiotic resistance of bacterial populations, but the secreted quantities must be assessed through offline measurements. Growth curve synchronization could be used to quantify the timing and quantity of indole production and help further elucidate the population dynamics. Our method could also be extended to include other online measurements such as pH quantification by color change of pH indicators (e.g. phenol red). Other endpoint measurements that can be integrated include direct measurements of gene expression (qRT-PCR or microarray analysis), quantifications of metabolite levels or protein quantities.

## Conclusions

The method of growth curve synchronization proposed here provides a simple, inexpensive solution to integrate rich time-resolved data with endpoint measurements. Like other model-based data integration methods [42], our method aims at a major limitation in systems biology -the scarceness of high quality time-resolved quantitative data. In the specific case of *P. aeruginosa*, this method can be used to validate and complement metabolic models. For example, the fluxes of secreted secondary metabolites measured for isogenic mutants can help further refine metabolic models from whole genome reconstruction [43,44]. Beyond *P. aeruginosa*, growth curve synchronization can be a general method to help unravel regulation dynamics in biological systems.

## Competing interests

The authors declare that they have no competing interests.

## List of abbreviations

μ_max _: maximum specific growth rate; λ_excitation _: excitation wavelength; λ_emission _: emission wavelength; τ: timeshift; 3O-C_12_-HSL: *N*-(3-oxododecanoyl)-L-homoserine lactone; C_4_-HSL: *N*-butyryl-L-homoserine lactone; GFP: green fluorescent protein; IND: strain with inducible rhamnolipid promoter; LB: lysogeny broth; NEG: rhamnolipid-deficient strain with GFP reporter fusion; OD_600 _: optical density at 600 nm; PBS: phosphate-buffered saline; PMN: polymorphonuclear leukocyte; rcf: relative centrifugal force; qRT-PCR: quantitative reverse transcription polymerase chain reaction; QSN: quorum sensing-negative strain with GFP reporter fusion; WT: wild-type strain with GFP reporter fusion.

## Authors' contributions

DD conceived the study, performed the experiments, analyzed and interpreted the data and wrote the paper. JXB conceived the study, wrote the alignment algorithm, interpreted the data and wrote the paper. All authors read and approved the final manuscript.

## Additional files

General comments

In order to run the Matlab demonstration (AdditionalFile3.m) place the two. csv files (AdditionalFile1.csv and AdditionalFile2.csv) in the same folder. Inside of this latter folder both of the .m files should be saved. The matlab code was written for Matlab R2010a with the statistics and optimization toolboxes.

## Supplementary Material

Additional file 1 **Matlab-based growth curve synchronization algorithm**. This is the main algorithm for growth curve alignment. The script calls AdditionalFile4.m and uses functions from the statistics and optimization toolboxes. The program draws plots of the data before alignment, after alignment, a time series of rhamnolipid production and the time shift versus dilution, yielding the growth rate. Click here for file

Additional file 2 **Matlab suite**. AdditionalFile4.m is a Matlab file implementing a suite of functions for reading, processing and plotting growth curve data. Click here for file

Additional file 3 **Raw data file for growth curve synchronization**. This file contains the raw data from a typical growth curve synchronization experiment. In this document, all the data is included, started with the optical density measurement (called od600) and then the GFP measurement (called gfp). Time is given in seconds. The first 8 samples (A1 through H1) are the blank, the second set of eight (A2 through H2) are from the culture inoculated at 0.0025 OD_600_, etc. The ninth set of eight (A9 through H9) contain the last set of data, the last sets (A10 through H12) are empty wells. This is one of the files used by the Matlab algorithm (AdditionalFile3.m) in order to synchronize the growth curves. Click here for file

Additional file 4 **Rhamnose quantification for different time points**. This file contains an example of rhamnose quantification from the sulfuric acid anthrone assay. The first column is from the sample inoculated at 0.0025 OD_600_, with subsequent dilutions for the following columns. The data is pre-processed for blank and averaged over four replicates, as well as normalized compared to a standard ladder of rhamnose. The first row is the average, the second row the maximal value and the third row the minimal value. This second file allows for the time series of rhamnolipids to be constructed. Click here for file

Additional file 5 **Excel-based growth curve synchronization**. Excel implementation of growth curve synchronization. Includes a spreadsheet ReadMe that explains the procedure. The included example uses the same data as the Matlab example. Click here for file
